# Colonization of patients hospitalized at orthopedic department of tertiary hospital in Uganda with extended-spectrum beta-lactamase-producing enterobacterales

**DOI:** 10.1186/s13756-023-01229-9

**Published:** 2023-04-01

**Authors:** Jules Bizimana, Jerome Ndayisenga, Henry Kajumbura, Phillip Mulepo, Najjuka Florence Christine

**Affiliations:** 1 Department of Medical Microbiology, Makerere University, Kampala, Uganda; 2 African Research and Community Health Initiative (ARCH Initiative), Kigali, Rwanda; 3 Orthopedic Department, Mulago National Referral Hospital, Kampala, Uganda

**Keywords:** Colonization of patients, Orthopedic Department, Mulago Referral Hospital, Extended-spectrum beta-lactamase producing Enterobacterales (ESBL-PE)

## Abstract

**Background:**

Beta-lactamase production remains the most contributing factor to beta-lactam resistance. Extended-Spectrum Beta-Lactamase-Producing Enterobacterales (ESBL-PE) are associated with risk factors both in hospital and community settings.

**Objectives:**

To assess the incidence and risk factors for intestinal carriage of ESBL-PE among patients admitted to orthopedic ward of Mulago National Referral Hospital, and to analyze the acquisition of ESBL-PE during hospital stay and associated factors.

**Methods:**

We screened 172 patients aged 18 years old and above who got admitted to the orthopedic ward of Mulago National Referral Hospital between May to July 2017. Stool samples or rectal swabs were collected at admission, every 3 days until fourteen days and screened for ESBL-PE. Data on demographic status, antibiotic use, admission and travel, length of hospital stay, hygiene practices and drinking boiled water were analyzed by logistic regression and cox regression model.

**Results:**

At admission, 61% of patients showed intestinal ESBL-PE carriage. Co- resistance was common but no Carbapenem resistance was detected. Of the ESBL-PE negative, 49% were colonized during hospitalization. On admission, prior antibiotic use was significantly associated with carriage, but none was associated with acquisition during hospitalization at p-value < 0.05.

**Conclusion:**

Carriage of ESBL-PE on admissions and acquisition at orthopedic ward of Mulago Hospital were high, and dissemination into the community are of substantial concern. We suggested refinement of empirical treatment based on risk stratification, and enhanced infection control measures that target health care workers, patients and attendants.

## Keywords

Colonization of Patients, Orthopedic Department, Mulago Referral Hospital, Extended Spectrum Beta Lactamase (ESBL-PE) Producing Enterobacterales.

## Background

Orthopedic surgical site infections are often associated with substantial morbidity and exorbitant costs, and are challenging to treat especially in case of multi-resistant pathogens or presence of implants [1]. In gram-negative pathogens, beta-lactamase production remains the most important contributing factor to beta-lactam resistance [2]. In the last decade, several studies have reported their spread to the community as well. Enterobacterales causes human infections such as gastrointestinal infections, septicemia, pneumonia, meningitis and urinary tract infections. Many clinical laboratories have reported that significant percentages of *Klebsiella* species and *Escherichia coli* infections acquired in the hospital setting are caused by strains that produce Extended-Spectrum Beta-Lactamase (ESBL) [3]. Beta–lactamase production is the most important defense mechanism against beta- lactam antibiotics. Beta lactamases include extended spectrum beta –lactamases (ESBLs), AmpC and carbapenemase. ESBLs confer resistance to penicillin, cephalosporin and Monobactam but are susceptible to Cephamycin and Carbapenems [4].

The risk factors associated with carriage of ESBL-PE are related to households of low income status, history of antibiotics use and history of admission in longer term care facilities both in hospital and community settings [5]. Similarly, use of antibiotics and history of admission in the last year have been reported to be independent risk factors for carriage of ESBL-PE among communities in Mwanza, Tanzania [6]. Some water sources and food of animal origin have been found to contain ESBL-PE [7]. Developed countries have also identified travel to countries where ESBLs are endemic to be a risk factor for acquisition of ESBL-PE [8]. The prevalence of ESBL-PE has been noted to be higher in developing countries where the standard of living is low such as Uganda [9].

Patients can acquire infection through the direct (hands) and indirect contacts, and also by coming into contact with the bacteria in the hospital or at home. Studies have shown interfamily spread of ESBL-PE and transmission has been observed to occur from companion and from animals to humans [7]. Septic wards are ideal place for the proliferation of resistant bacteria due to the selective pressure exerted by intensive use of penicillin and cephalosporin, long hospital stays with intensive close nursing and physiotherapy for multi-morbid and immobile patients, high prevalence of open wounds, ulcers or external fixation devices, and lack of established decolonization protocols of ESBL-PE [1].

Carriage of ESBL-PE remains high among outpatient clinics in Kampala [10]. Wards cohorting infected orthopedic patients may be particularly prone to transmitting ESBL-PE. These infections are often associated with substantial morbidity and exorbitant costs, and are challenging to treat, especially in case of multi-resistant pathogens or presence of implants [1]. Despite the fact that a number of studies have been done to provide insights in regards to the prevalence of ESBL-PE at the hospitals, limited data exists about the risk factors associated with acquisition of ESBL-PE on admission and during hospitalization at Mulago National Referral Hospital. This study aims to determine the proportion of patients carrying ESBL-PE on admission, the proportion of patients who acquire ESBL-PE during the period of hospitalization, to identify the risk factors associated with carriage ESBL-PE from the community and acquisition of ESBL-PE during the period of hospitalization, with a major focus on the orthopedics ward at Mulago National Referral Hospital.

## Methods

### Study design and population

This was a longitudinal study which was conducted from May 2017 to July 2017 at Mulago National Referral Hospital, a tertiary hospital in Uganda. The subjects were 172 adult’s patients (18 years and above) admitted with trauma on the orthopedic wards at Mulago National Referral Hospital.

### Study site and setting

The study was conducted at Mulago hospital, orthopedics wards and the collected samples were processed in the Clinical microbiology laboratory under the college of Health Sciences, Makerere University.

Mulago hospital is the main National Referral hospital of Uganda and serves as a teaching hospital for the Makerere College of Health Sciences and is a General Hospital of Kampala, Uganda. Mulago Hospital Orthopedic ward is a specialized surgical unit, with different sections such as trauma, spinal and children’s units. The unit has two major operating theatres one for trauma patients and the other for spinal patients. It has a bed capacity of over 100–120 beds, and receives on average 10 cases each day. Majority of these admitted cases are for surgery and their average hospitalization period is 30 days. The unit is boosted with a number of surgeons, nurses, and orthopedic offices among others. The collected stool or rectal swab samples were processed in the Medical Microbiology Department of Makerere University that acts as teaching and research laboratory, and participates in Proficiency Testing (PT) of the American College of Pathologist (CAP No 732255-93-01).

### Data collection

Sampling was consecutive sampling by choosing the first 172 patients admitted on the trauma orthopedic wards upon fitting in the inclusion criteria. A questionnaire was developed after reviewing different literature on factors associated with colonization with ESBL-PE [11–16]. A pretested questionnaire was used to collect information on demographic characteristics, history of admission, history of antibiotic use, travel and care of animals, hygiene practices and antibiotics used in the hospital, demographics, history of antibiotic use, history of admission, travel and care of animals were measured as independent variables whereas EBLs, AmpC and Carbapemase resistance were considered as dependent variables.

### Laboratory testing

#### Fecal sample collection, preparation and storage

The samples collection procedures were explained to the patients and advised to collect a fresh stool sample while avoiding any contamination and delivering to the study staff within four hours of collection. Sterile, dry screw-top containers were used for stool samples collection. When the patient failed to provide stool, a rectal swab was given to him/her to collect fecal matter by moving the swab from front to back. The specimens were labeled and transported from the wards to the Department of Medical Microbiology Laboratory, School of Biomedical Sciences, College of Health Sciences, Makerere University. Pure Gram-negative isolates were preserved in Brain Heart Broth (BHI) containing 30% of glycerol and stored in -80 °C in the freezer for further analysis in future.

#### Identification of bacteria and initial screening for ESBL

Isolation of bacteria from Stool samples was achieved as described. Rectal or stool samples were suspended into an isotonic solution of phosphate buffered saline (PBS), and this was followed by vortexing until the sample was completely suspended. The suspension was then inoculated onto MacConkey agar supplemented with Cefotaxime (2 µg/ml) for preliminary screening of ESBL-PE, incubation of these cultures was done aerobically at 35–37°c. Bacterial growth from these cultures were checked after 24 h. All gram- negative bacteria that had growth were identified by using colony characteristics on MacConkey agar, and by using Biochemical tests namely Urease, citrate, Sulphur indole, Motility (SIM) and Triple sugar iron (TSI) test.

#### Antimicrobial susceptibility testing

Antimicrobial susceptibility testing procedures were performed using the Kirby- Bauer Disc diffusion method on Mueller Hinton agar (MHA) plates according to the Clinical and Laboratory Standard Institute (CLSI) guidelines [17]. Antimicrobial disks that were used are: Ceftazidime (CAZ), Cefotaxime (CTX), Cefepime (FEP), Cefoxitin (FOX), Meropenem (MEM), Amoxicillin –clavulanate (AMC), Temocillin (TEM), Piperacillin –tazobactam (PTZ), Chloramphenicol (C), Gentamicin (GEN), ciprofloxacin (CIP), Tetracycline (TE), Ceftriaxone (CRO), Aztreonam (ATM), and Sulfamethoxazole (SUL).

#### Confirmation of ESBLs

Confirmation of ESBLs was done by combined disc method. Isolates of 0.5 McFarland were streaked on Mueller-Hinton Agar (MHA) plates, discs placed (CTX, CAZ, FEP, CRO, ATM) and incubated in ambient air. ESBLs presence was confirmed by demonstration of synergy between cephalosporin and clavulanic acid. Increase of ≥ 5 mm inhibition halo of disks containing cephalosporin plus clavulanic acid as opposed to disks with cephalosporin alone confirmed ESBL-producing organisms.

#### Detection of AmpC

Detection of AmpC enzymes was done by use of disc diffusion testing with Cefoxitin (FOX) alone/ Cefoxitin with cloxacillin (FOX/ FOX + CLOX). Disks were placed on Mueller Hinton agar plate, which was inoculated with test strain. Incubation was done overnight. If the difference between the combination discs of FOX + CLOX and disc of cefoxitin (FOX) alone is ≥ 4 mm, it indicated a positive result for AmpC enzyme production.

#### Detection of carbapenemase producers

Modified Hodge Test with disc of Meropenem (10ug) was used to detect carbapenemase production on MHA plates. Carbapenemase inhibition method was used to screen both *Klebsiella pneumoniae* Carbapenemase (KPC) and Metallo β-Lactamases (MBLs) production in the bacteria. In the Detection of MBLs, one disc of meropenem alone, and a combination disc of Meropenem with EDTA (Mem /Mem + EDTA) were placed on MHA inoculated with a bacterial suspension of 0.5 McFarland and incubated overnight. Metallo beta- lactamases (MBLs) producing strains showed a variation ≥ 5 mm between the inhibition zone around Meropenem alone and Meropenem with EDTA. In detection of KPC, meropenem disc alone, and meropenem disc with combination of Boronic acid was used, if a variation ≥ 4 mm between zone around Meropenem alone and Meropenem with boronic acid disc (Mem /Mem + boronic acid) noted, it indicated a KPC producer.

Indicator strains, *E. coli* ATCC 25,922 and *K. pneumoniae* ATCC700603 were used as indicator organisms in ESBL screening and *Klebsiella pneumoniae* ATCC BAA 1705 and ATCC BAA 1706 were served as positive and negative control strains for MHT. Testing procedures followed Clinical and Laboratory Standard Institute (CLSI) guidelines [17].

### Data analysis

The data collected was double entered into EPIDATA version 3.1 for validation. It was then exported to STATA version 13 for further management and for analysis. Data were analyzed using descriptive and analytical statistics. Univariate analysis was done for descriptive analysis. Chi-square test and t-test were used to test association between a patient’s characteristics with ESBL-PE carriage at admission and during hospitalization, and significance deliberated at P-value less than 0.05.

We used Kaplan-Meier Survival Estimate to find median time of getting ESBL-PE infection for the patients who were admitted with no ESBL-PE infection. The proportion of patients that acquire ESBL-PE after admission and the median time to getting ESBL-PE were reported. T-test and ANOVA were used to compare means and medians of continuous (respectively normally and non-normally distributed) variables across ESBL-PE status levels after admission. A Cox proportional hazards model was used to determine both the unadjusted and adjusted hazard ratios.

## Results

### Demographic and baseline characteristics of study participants

The study recruited 172 participants that had been admitted with a history of trauma at Mulango National Referral Hospital Orthopedic Department. The majority 127 (73.8%) were male, and 158 (92%) of the participants were less than 60 years of age. Out of the total participants recruited, 88 (51.2%) were married, 67 (38.9%) were single, 12 (7.0%) were divorced, and 5 (2.9%) were widows. In regard to place of residence, the majority 108 (62.8%) of the patients were town dwellers.

**Table 1:** Baseline characteristics of study participants

Water, Hygiene and Patient History characteristics at admission and during hospitalization of participants	
Characteristic	Frequency
**Drinking boiled water**	
Yes	142(83.0)
No	30(17.0)
**Hand washing (after toilet)**	
Yes	147(85.5)
No	25(14.5)
**Hand washing (before food)**	
Yes	167(97.1)
No	5(2.9)
**Use of soap for Hand washing**	
Yes	13(7.6)
No	159(92.4)
**Previous hospital Admission (3–12 months)**	
Yes	12 (7.0)
No	160 (93.0)
**Used antibiotics in 3 or 12 months**	
Yes	41 (23.8)
No	131 (76.2)
**Keep animals**	
Yes	64(37.2)
No	108(62.8)
**Travel History (3 or 12 months)**	
Yes	35(20.3)
No	137(79.7)
**Median days of hospital stay (IQR)**	1 (1,3)

The Table 1 above shows that more than half of the participants consume boiled water (83%). A majority of the participants did not use soap to wash hands 92% (159). The patients without a recent admission history were 159 (93.0%). Findings showed a 76.6% (131) prior use of antibiotics during the last 3 or 12 months. Other characteristics are summarized in Table 1 above.

### Proportion of ESBL-PE, isolated bacterial species and results of antimicrobial susceptibility testing

Of the 172 participants, 61% (105) had ESBLs on admission, 2% (3) were positive for AmpC and none had carbapenemase. A total of 142 isolates were found from the fecal samples, and the most prevalent were *E. coli* 108 (76.1%), followed by *Klebsiella spp*. 23 (16.2%), *Citrobacter spp*. 5 (3.5%), *Acinetobacter spp*. 4 (2.8%), and the least were *Enterobacter spp*. 2 (1.4%). Both ESBLs and AmpC were detected in *E. coli* only. Antimicrobial susceptibility testing showed that isolates were most susceptible to Meropenem (97.1%) and least susceptible to Cefotaxime (2.1%). The results of antimicrobial susceptibility testing are presented in Table 2 below that shows the susceptible proportion.

**Table 2:** Antimicrobial susceptibility profile of the ESBL-producing Enterobacterales isolates showing the susceptible proportion

Antibiotic	Total isolates (N = 142) n (%)	*E. coli (*N = 108) n (%)	*K.pneumoniae* (N = 23) n (%)	*Citrobacter spp (*N = 5) n (%)	*Acinetobacter spp (*N = 4) n (%)	*Enterobacter spp (*N = 2) n (%)
Meropenem	138 (97.2)	107 (99.1)	21 (91.3)	5 (100.0)	3 (75.0)	2 (100.0)
Amoxicillin-Clavulanate	34 (23.9)	32 (29.6)	2 (8.7)	0 (0.0)	0 (0.0)	0 (0.0)
Ceftriaxone	9 (6.3)	1 (0.9)	4 (17.4)	1 (20.0)	2 (50.0)	1 (50.0)
Piperacillin-tazobactam	77 (54.2)	48 (44.4)	20 (87)	4 (80.0)	3 (75.0)	2 (100.0)
Cefepime	30 (21.1)	19 (17.6)	5 (21.7)	4 (80.0)	2 (50.0)	0 (0.0)
Ciprofloxacin	43 (30.2)	24 (22.2)	14 (60.9)	3 (60.0)	1 (25.0)	1 (50.0)
Gentamycin	85 (59.9)	60 (55.5)	15 (65.2)	5 (100.0)	4 (100.0)	1 (50.0)
Cefoxitin	74 (52.1)	57 (52.8)	14 (60.9)	0 (0.0)	1 (25.0)	2 (100.0)
Ceftazidime	6(4.2)	2 (1.8)	2 (8.7)	0 (0.0)	2 (50.0)	0 (0.0)
Cefotaxime	3 (2.1)	0 (0.0)	3 (13.0)	0 (0.0)	0 (0.0)	0 (0.0)
Aztreonam	21 (14.8)	5 (4.6)	8 (34.8)	2 (40.0)	4 (10.0)	2 (100.0)
Temocillin	100 (70.4)	79 (73.1)	20 (87)	1 (20.0)	0 (0.0)	0 (0.0)
Chloramphenicol	85 (59.9)	61 (56.5)	18 (78.2)	5 (100.0)	0 (0.0)	1 (50.0)
Tetracycline	18 (12.7)	7 (6.5)	6 (26.1)	3 (60.0)	2 (50.0)	0 (0.0)
Sulfamethoxazole	24 (16.9)	4 (3.7)	12 (52.2)	4 (80.0)	4 (100.0)	0 (0.0)

### Risk factors of carrying ESBL-PE on admission to hospital

Bivariate analysis of risk factors associated with carrying ESBL-PE at admission to Mulago hospital was done and significance association deliberated at P-value < 0.05, and the results are presented in Table 3 below. Prior antibiotic use in the past 3–12 months was found to be significantly associated with ESBL-PE carriage at admission RR 2.6 95% CI (1.16-6.00), P-value of 0.020. Travel history and prior admission to the hospital showed no statistically significant association with ESBL-PE carriage on admission (RR 1.7 CI (0.81–3.63) and RR 0.2 CI (0.002–1.37) respectively).

**Table 3:** Bivariate Logistic regression analysis of risk factors of carrying ESBL-PE at admission to Mulago hospital

Characteristic	Risk Ratio	95% (CI)	p-value
**Sex**			
Male	1		
Female	0.9	(0.43–1.73)	0.672
**Age**			
18-59years	1		
> 60years	1.6	(0.48–5.35)	0.441
**Drinking boiled water**			
Yes	1		
No	1.3	(0.56–2.97)	0.543
**Hand washing (toilet)**			
Yes	1		
No	0.8	(0.35–2.04)	0.709
**Hand washing (food)**			
Yes	1		
No	0.9	(0.15–5.70)	0.934
**Prior antibiotic use**			
No	1		
Yes	2.6	(1.16-6.00)	0.020
**Travel history**			
**Yes**	1		
No	1.7	(0.81–3.63)	0.160
**History of Admission**			
Yes	1		
No	0.2	(0.002–1.37)	0.096

### Proportion of patients acquired ESBL-PE during hospitalization

The total proportion of patients carrying ESBL-PE during hospitalization was 49% (26/53). Median time of getting a positive ESBL-PE result was 3 days (IQR: 1–3). The first events were observed soon by day 3 and they gradually decreased up to the 15th day. The proportion increased with the length of stay in hospital. Among enrolled study participants, 67 (39%) were negative of ESBL-PE on admission, and they were followed for infection during hospitalization. On Day 3, 53 participants remained in hospital settings and participated in the study, and among them, 12 (23%) were positive with an incidence rate/100 person days of 20.2. On day 6, 28 participants remained and participated in the study, and 8 (29%) were positive with an incidence rate of 7. On day 9, 12 participants remained and participated in the study, and 4 (33%) were positive with an incidence rate of 3.5. On day 12, 6 participants remained and participated in the study, and none was positive among them. On day 15, 6 participants remained and participated in the study, and 2 (33%) were positive with an incidence rate of 1.8.

### Risk factors associated with acquisition of ESBL-PE during hospitalization at Mulago

Risk factors of carrying ESBL-PE during hospitalization at Orthopedic ward of Mulago hospital were analyzed by bivariate proportional hazard cox regression model, and results are presented in Table 4 below. No variable was found to be statistically associated with ESBL-PE carriage at P-value less than 0.05. Length of hospital stay showed no statistically significant association with ESBL-PE carriage at RR 0.3 95% CI (0.55–1.01) and P-value 0.059.

**Table 4:** Bivariate proportional hazard cox regression model analysis of risk factors of carrying ESBL-PE during hospitalization at Orthopedic ward of Mulago hospital

Characteristic	RR	95% (CI)		P-value
**Length of hospital stay**	0.3	(0.55–1.01)		0.059
**Sex**				
Male	1.0			
Female	0.8	(0.27–2.08)		0.587
**Handwashing after toilet**				
Yes	1.0			
no	0.9	(0.27–3.13)		0.886

## Discussion

We carried out a prospective study to determine the proportion of ESBLPE and risk factors associated with carriage among patients at admission and during hospitalization in orthopedic wards at Mulago national referral hospital. This study found a high proportion of ESBL-PE at admission (61%), but also high numbers during hospitalization (49%). Colonization with ESBL-PE is a risk for ESBL-PE infections in developing countries which are associated with delays, ineffective treatment, increased cost, morbidity and mortality [15].

### Prevalence of ESLB-PE at admission and during hospitalization

The current study showed prevalence of ESBL-PE at admission of 61% while the prevalence during hospitalization was 49%. Findings are in agreement with a study done in Sub Saharan Africa that found ESBL-PE in hospital and community settings above 50% [18]. A study done in Rwanda found a high intestinal ESBL-PE carriage (50%) among admitted patients from the community and acquired ESBL-PE (55%) at discharge [19]. The highest rate of ESBL-PE fecal carriage was reported in Egypt (63.3%) [20]. The prevalence at admission in this study is a good indicator of prevalence in the community. However, this study findings are in contrast with an ESBL-PE carriage of 11.6% reported in developed High Income Countries (HICs) [21]. The reason for this disparity is because of the difference in hygiene and sanitation settings. The high prevalence during hospitalization is an indicator of nosocomial infections, which highlights calls for infection control during hospitalization.

Hospital transmission could be associated with hygiene and sanitation factors of the patient, health worker, and caregiver. The community transmission witnessed by high prevalence at admission can be explained by Intra household transmission of ESBL-PE preceding hospitalization to health facilities and shared prior exposure might have exerted parallel acquisition of ESBL-PE among patients at admission [22]. This is further supported by the fact that the majority of the participants did hand washing before meals and after toilet use, but a big number (92.4%) were not using soap.

In our study, the majority of the enrolled and affected study participants in the orthopedic wards were males compared to females. The reason could be the fact that males are more exposed to the trauma mainly due to their nature of work which puts them at bigger risk. In Kampala, where the study site is located, the majority of orthopedic cases arise out of transport accidents mainly motorcycle accidents and men are the main people involved in this trade.

### Risk factors for ESLB-PE carriage at admission and acquisition during hospitalization

The study identified prior antibiotic treatment as a risk factor associated with ESBL-PE carriage at admission RR 2.6 95% CI (1.16-6.00), P-value of 0.020. Patients who had been treated on antibiotics prior to admission were more likely to have ESBL-PE carriage. This has been found as a big risk factor in previous studies [5]. In Uganda and other similar African settings, there is a lot of irrational drug use in various forms including self-prescriptions, poor dosing, incomplete treatment and use of antibiotics for conditions not warranted. This could explain the link between prior antibiotic use and acquisition of ESBL-PE [23].

The study found that the colonization of ESBL-PE increased for patients who stayed in the hospital for more than 6 days as compared to those who stayed between 1 and 3 days though it was not statistically significant. Patients who stayed longer than six days had a higher chance of ESBL carriage, and it is explained by the increased exposure associated with long hospital stays. The current study found that length of hospital stay was associated with carriage of ESBL-PE during hospitalization; However, this relationship was not statistically significant at p-value of < 0.05. Whereas previous studies have found hand washing after using the toilet and length of hospital stay as factors associated with ESBL-PE acquisition [24], no significant association was found in this study. This lack of significance could be caused by the small sample size for the hospitalized patients.

In this study, we found that Meropenem was more sensitive than any other antibiotics to treat ESBL-PE. In Uganda, congruent results were found in a study conducted by Najjuka et al. [10], in which Meropenem was the antibiotic of choice of ESBL-PE infection. However, the high costs of carbapenems limit their availability at limited resources. Other antibiotics including third generation cephalosporins like ceftriaxone, ceftazidime, etc. were more resistant; hence, not recommended for treatment of ESBL-PE infection in study setting.

A total of 142 isolates were tested for meropenem susceptibility and 138 were susceptible. All isolates were tested for carbapenemase production, and no carbapenem producing enterobacterales identified. This confirms that reasons for reduced sensitivity to meropenem for the remaining four isolates are associated with other factors apart from carbapenemase production. Existing literature highlight that other factors that may cause resistance to carbapenem include alteration of cell membrane porin channels, efflux pump, and target mutation [25, 26]. While carbapenemase production is the main cause of carbapenem resistance, a recent study conducted in Texas found that among all carbapenem resistant enterobacterales, 59% were non-carbapenem producing enterobacterales. This study conducted in Texas also identified non-carbapenemase producing k. pneumoniae failure to meropenem therapy, which was associated with OmpK36 mutation causing decreased intracellular accumulation of antibiotics [27].

### Study limitation

We had participants that lost follow-up for 14 days because of early and self-discharge from the hospital. Patients available and not available for follow up had similar baseline characterization, and this could have neutralized any major impact of loss to follow up on estimated rate of ESBL-PE acquisition during hospitalization.

## Conclusion

In this study, the extent of ESBL-PE acquisition at the orthopedic ward of Mulago Hospital was high at both admission (61%) and hospitalization stages (49%). Prior use of antibiotics increased the risk of ESBL-PE carriage at admission. The incidence of ESBL-PE carriage during hospitalization increased with longer hospital stay. Mulago National Referral Hospital could improve the capacity of health workers in diagnostic capacities, routine admission screening for ESBL-PE carriage among patients and also during hospitalization. The ministry of health could develop guidelines for management and screening for ESBL-PE. The level of Multidrug resistance calls for strengthening of laboratories to be able to detect these varied patterns of bacterial isolates causing infection so as to guide therapy. The acquisition of ESBL-PE during admission calls for enhanced infection control measures that target both health care workers and patients plus their attendants. The communities should be sensitized on antimicrobial resistance as a result of inappropriate use of antibiotics.

## Data Availability

The dataset used in this study can be made available upon reasonable request to corresponding author.
